# Regulating the unseen hand: AI, authorship, and trust in medical science

**DOI:** 10.1097/MS9.0000000000003498

**Published:** 2025-06-16

**Authors:** Naveen Gautam

**Affiliations:** Maharajgunj Medical Campus, Institute of Medicine, Tribhuvan University, Kathmandu, Nepal

**Keywords:** AI regulation, artificial intelligence, authorship, large language models, medical ethics

## Abstract

Large language models (LLMs) have transformed medical research and scientific publishing by facilitating manuscript preparation, literature synthesis, and editorial processes, yet pose significant threats to research integrity through generation of potential pseudoscientific content. Current AI detection algorithms demonstrate inconsistent reliability, particularly against paraphrased or humanized content, while LLM integration in peer review compromises expert critical evaluation and homogenizes scientific discourse. These systems exhibit documented bias against non-male, non-white researchers, compounding ethical concerns. Heterogeneous editorial policies regarding AI disclosure across medical journals create regulatory gaps enabling undetected misconduct. However, excessive focus on detection over content quality risks establishing counterproductive "AI phobia" that impedes legitimate technological integration. Preserving research credibility requires standardized disclosure frameworks, enhanced detection algorithms, comprehensive privacy safeguards, and mandatory AI watermarking systems to maintain scientific integrity while accommodating technological advancement in research practices.

Large language models (LLMs) have transformed medical research and publishing, with ChatGPT reaching 100 million users by 2 months of its release^[^1^]^. A 2024 survey showed that 76% of researchers integrate artificial intelligence (AI) tools in their workflows because they expedite the process of drafting manuscripts, reviewing literature, correcting grammar, and promoting scientific equity for non-native English speakers^[^2,3^]^. This utility masks another threat that comes with the ease of using them. Such tools can easily generate content that appear scientifically plausible but in the core are fundamentally fraudulent and fabricated, threatening research integrity. An example of this duality was demonstrated in a 2023 article where a series of 13 prompts were given to ChatGPT to produce a manuscript for publication in PLOS Medicine. The prompt generated a 1992-word manuscript on neurosurgery with a title, introduction, results, discussion, abstract, and 17 citations in merely an hour^[^3^]^. While polished, expert reviewers identified inadequate exclusion criteria, inconsistent statistical data, implausible clinical outcomes, and eight flawed citations^[^3^]^. This tension between utility and potential misuse calls for robust regulatory guidelines to safeguard the basic tenets of scientific research. The increasing integration of AI into scholarly work necessitates clear guidelines for transparency in its reporting. Recognizing this need, the TITAN guidelines offer a framework for the transparent reporting of AI in research manuscripts^[^4^]^.

**HIGHLIGHTS**

- The current unreliability of AI detection tools necessitates a multi-faceted approach beyond simple detection.
- Skepticism toward AI’s role in critical peer review processes is warranted due to high inaccuracy rates and lack of nuanced reasoning.
- Non-uniform editorial policies on AI use create vulnerabilities for scientific misconduct.
- Mandating linked references (DOIs/weblinks) can be an immediate step to combat citation fraud.
- A crucial balance must be struck between leveraging AI’s benefits and mitigating its threats through robust regulation, avoiding an “AI phobia” that stifles genuine innovation. Without regulation, AI risks becoming an “unseen hand,” an uncredited co-author eroding the credibility of scientific writing. The urgency for comprehensive reform, including standardized disclosure policies, advanced detection methods, ethical AI training, and robust data verification, is clear-scientific integrity hangs in the balance.

Currently, detection tools struggle to reliably detect AI-generated content, especially when they are rephrased^[^1^]^. Traditional plagiarism detection tools, such as Urkund, for instance, find low similarity scores (around 23%) for AI-rephrased articles^[^1,5^]^. Even specialized detection tools show variable performance. One study showed that Originality.ai claimed 100% detection of rephrased articles, ZeroGPT at 88%, while Turnitin flagged only 30%^[^5^]^. Another tool, BrandWell AI Writer and Editor Review (formerly Content at Scale) assigned a modest 48% probability to a known AI-authored article^[^3^]^. This inconsistency shows a critical gap where it becomes extremely difficult to identify AI-generated text as researchers may copy LLM verbatim outputs in their writing, assuming them undetectable.

AI’s potential role in peer review is even more alarming. While a minority (11%) of researchers value its time-saving efficiency 6), a significant proportion (44%) find chatbots unhelpful, with a majority (67%) rejecting it for critical assessments such as research analysis, interpretation, and decision-making^[^6^]^. This skepticism is justified, as 38–48% of AI-generated review comments are highly inaccurate, often lacking critical reasoning and methodological rigor that are foundational to modern scientific inquiry^[^6^]^. Additionally, LLMs pose a risk of homogenizing scientific discourse to the extent it fails to portray inherent heterogeneity in scientific perspectives^[^7^]^. Moreover, studies have shown that AI generated texts can exhibit bias against non-male, non-white scientists^[^2^]^ adding further ethical complexity to its use.

Further complicating this ethical landscape is the non-uniform editorial policies across medical journals. Analysis of the top 100 journals show significant variation in AI-use disclosure: 78% offer guidance on utilization, 59% ban it, whereas 41% permit limited, regulated use^[^8^]^. Confidentiality concerns are nearly universal (96%). About 91% of journals prohibit manuscript uploads to AI systems due to concern of data breaches^[^8^]^. Such inconsistent editorial policies across journals heighten the risk of undetected misconduct, as AI tools can generate fabricated but convincing discussions and citations^[^9^]^.

An effective way to combat citation fraud can be to mandate researchers to include all references together with their weblinks or DOIs. This would allow for immediate verification of cited sources, discouraging the use. However, detecting AI-generated discussions is particularly challenging, as they often appear well-structured and plausible while subtly distorting scientific narratives. This challenge is amplified by the use of AI text humanizers, which modify texts to evade detection further complicating this cat-and-mouse game. Conversely, focusing solely on AI text detection rather than the content quality can shift focus away from the core scientific substance creating an “AI phobia” in medical research. Therefore, seeking a crucial balance is essential.

Strict regulatory measures are non-negotiable to address these threats. Standardized AI-use disclosure policies, advanced detection algorithms, robust privacy measures, and systematic data verification could mitigate fraud risks. AI watermarking and mandatory ethical AI training are vital to balance innovation with integrity^[^10^]^. Without regulation, AI risks become an “unseen hand,” an uncredited co-author eroding credibility of scientific writing. Medical research stands at a crossroads: AI’s integration offers transformative potential, yet its unchecked proliferation jeopardizes the principles of evidence-based medicine. The urgency for reform is clear – scientific integrity hangs in the balance.

## Data Availability

Data sharing is not applicable to this article as no new datasets were generated or analyzed during the current study. All information is based on publicly available cited literature.

## Declaration on the use of AI in manuscript preparation

Generative AI tools (Gemini 2.5 Pro, Google) were utilized in the preparation of this manuscript solely for grammar correction. All AI-generated suggestions were critically reviewed, edited, and verified by the authors. The remainder of the manuscript, including all content, research, and drafting, was completed by the human authors. The final responsibility for the content of this manuscript rests entirely with the human authors. The supplementary file lists the details, http://links.lww.com/MS9/A854.
